# Psychometric validation of the Loyola Generativity Scale in Chilean teachers: evidence for a three factor structure

**DOI:** 10.3389/fpsyg.2026.1913448

**Published:** 2026-08-26

**Authors:** Eduardo Sandoval-Obando, Alejandro Vega-Muñoz, Mauricio Véliz-Campos, Luis Castellanos-Alvarenga, Hernán Riquelme-Brevis, Dante Castillo

**Affiliations:** 1 Facultad de Ciencias Sociales y Humanidades, Universidad Autónoma de Chile, Temuco, Chile; 2 Facultad de Medicina y Ciencias de la Salud, Universidad Central de Chile, Santiago, Chile; 3 Centro de Investigación en Medicina de Altura, Universidad Arturo Prat, Santiago, Chile; 4 Facultad de Educación, Universidad de Talca, Talca, Chile; 5 Facultad de Ciencias Sociales y Educación, Escuela de Psicología, Universidad Santo Tomás, Temuco, Chile; 6 Coordinación General de Investigación y Proyección Social, Instituto Especializado de Profesionales de la Salud, San Salvador, El Salvador; 7 Facultad de Humanidades, Universidad de Santiago de Chile, Santiago, Chile

**Keywords:** educational psychology, professional development, psychometric assessment, socioemotional competence, teacher generativity

## Abstract

This study examined the psychometric properties and factorial structure of the Loyola Generativity Scale (LGS) in a large sample of Chilean primary school teachers, addressing the need for validated measures of generativity in Latin American educational contexts. Using a cross-sectional design and a rigorous analytical framework that included item purification based on MSA criteria, polychoric correlations, robust unweighted least-squares estimation, and a Solomon split-sample approach, the findings provide strong evidence for the reliability and structural validity of a refined version of the scale. Confirmatory factor analysis supported a theoretically meaningful three-factor structure composed of Generative Inefficacy, Generative Action, and Generative Legacy. Generative Inefficacy reflected reduced perceptions of generative effectiveness and personal impact. Generative Action captured concrete prosocial behaviors and commitments associated with teaching, creativity, productivity, and community involvement, and Generative Legacy represented motivations related to symbolic continuity and contribution to future generations. The correlations among factors indicated related but distinct dimensions, supporting a multidimensional understanding of generativity in the teaching profession. Reliability indicators demonstrated strong internal consistency and high construct replicability. Descriptive findings showed consistently high levels of generativity across demographic groups, reinforcing theoretical perspectives that view teaching as a profession intrinsically oriented toward supporting future generations. These results are consistent with previous research linking generativity to professional identity, prosocial motivation, socioemotional functioning, and intergenerational contribution. The validated instrument provides a solid basis for future studies examining the role of generativity in teacher well-being, professional development, pedagogical engagement, school climate, and educational outcomes. Future research should evaluate measurement invariance across relevant groups, explore changes in generativity throughout teachers’ careers, and incorporate this construct into broader explanatory models of teacher development and educational effectiveness. Overall, the study strengthens the assessment of generativity in Latin America and highlights its importance as a valuable psychological resource within the teaching profession.

## 1 Introduction

Generativity, the enduring concern for contributing to the well-being, development, and continuity of future generations, has re-emerged as a central construct for understanding teachers’ professional identity, motivation, and psychological functioning. Rooted in Erikson’s psychosocial theory, generativity has traditionally been conceptualized as a midlife developmental task (Van De Water and McAdams, 1989). Yet contemporary research across education, psychology, sociology, and learning sciences demonstrates that generativity is not merely a stage-bound disposition but a core dimension of teaching as a vocation, a driver of pedagogical commitment, and a predictor of well-being and professional flourishing. This expanded view positions generativity not as a peripheral trait but as a central organizing construct for understanding teachers’ work, identity, and development. Validating the Loyola Generativity Scale (LGS; McAdams and de St. Aubin, 1992) among Chilean teachers is therefore not only psychometrically necessary but also theoretically urgent: it enables the field to measure a construct increasingly recognized as foundational to the teaching profession.

Across diverse educational contexts, generativity appears as a motivational, relational, and identity-based force that shapes how teachers interpret their work and engage with students, colleagues, and communities. Studies with secondary teachers show that emotional intelligence profiles predict higher positive generativity and lower generative doubts (Aparisi et al., 2020), suggesting that generativity is intertwined with socio-emotional competencies essential for teaching. Research with rural educators highlights generativity as a source of resilience, community engagement, and subjective well-being, especially in resource-limited environments where teachers’ work is deeply embedded in local social fabrics (Gómez-Domínguez et al., 2025). Similarly, qualitative studies with veteran rural teachers reveal relational dynamics of trust, reciprocity, and leadership that reflect a mature generative identity oriented toward community development and intergenerational continuity (Sandoval-Obando et al., 2022). These findings converge on a central insight: teaching is inherently generative, and generativity is a key psychological resource sustaining teachers’ commitment and sense of purpose.

Generativity also emerges as a critical dimension of teacher well-being. A recent scoping review shows that generativity strengthens teachers’ sense of purpose and pedagogical engagement, while psychological well-being modulates resilience and instructional effectiveness (Sandoval-Obando et al., 2025). This aligns with broader developmental research demonstrating that community contribution, a generative value, is strongly associated with vitality among teachers, more so than self-growth values typically emphasized in younger adults (Lekes et al., 2016; Franco-López et al., 2020; Sandoval-Obando et al., 2025). Together, these findings underscore that generativity is not only a professional disposition but a psychological anchor that supports teachers’ mental health, motivation, and long-term engagement.

Beyond individual psychology, generativity is increasingly recognized as a pedagogical and epistemic resource. In science education, teachers’ attunement to the “intellectual generativity” of students’ sense-making – seeing students’ ideas as productive rather than deficient – has been shown to expand teachers’ interpretive power and foster more equitable, expansive pedagogical practices (Rosebery et al., 2016). Similarly, research on learning progressions reveals that even when models are empirically imperfect, teachers use them generatively – as starting points for inquiry, hypothesis testing, and instructional innovation (Alonzo and Elby, 2019). Generativity thus describes not only teachers’ concern for future generations but also their capacity to generate new knowledge, practices, and interpretations in dynamic instructional contexts.

Generativity also appears in practice-embedded teacher learning. Studies of coaching interactions show that brief, cumulative exchanges during instruction can produce generative pedagogical sensemaking, enabling teachers to move across within-, across-, and beyond-lesson perspectives (Munson and Dyer, 2023). Similarly, participatory action research with music educators demonstrates that identity formation is recursive, dialogic, and generative, emerging through collaborative risk-taking and co-construction (Weatherly and Coles, 2026). These findings reinforce that generativity is not merely a trait, but a dynamic process enacted through discourse, collaboration, and reflective practice.

The construct also extends into intergenerational and community-based educational settings. Intergenerational childcare programs enhance older adults’ sense of purpose, self-worth, and social connection, and classic generative outcomes, while improving classroom environments for younger teachers (Newman and Riess, 1992). Place-based stewardship education fosters students’ generativity by cultivating awareness of ecological interdependence, collective efficacy, and the legacy of environmental action (Flanagan et al., 2019). In cultural and artistic domains, generativity appears in the transmission of embodied knowledge, as in flamenco communities where elders are valued as bearers of moral and sentimental wisdom (Soria, 2024), or in decolonial pedagogies that emphasize embodied generativity as a mode of resisting colonial epistemologies (Kaktikar, 2020). These examples illustrate that generativity is deeply embedded in cultural, communal, and intergenerational practices, reinforcing its relevance for educational research.

Generativity also intersects with narrative identity and meaning making. Narrative theory highlights generativity as a central dimension of how individuals construct life stories that integrate past, present, and future (McAdams, 2001). Autobiographical studies show that generativity is predicted by positive socializing influences from family, teachers, and institutions, though moderated by socioeconomic and racial factors (Jones and McAdams, 2013). In early childhood education, narrative practices that emphasize creativity and generativity foster children’s subjectivity, intersubjectivity, and cultural learning (Jiang et al., 2026). In philosophical perspectives, generativity appears in conceptions of self-awareness that grapple with uncertainty, mortality, and the ethical responsibility toward future generations (Sevilla-Liu, 2021). Across these traditions, generativity emerges as a temporal, narrative, and ethical construct that links identity formation with responsibility toward others.

Even in emerging fields such as artificial intelligence in education, generativity is a contested and illuminating concept. Technical definitions of generativity in machine learning contrast with psychosocial views that emphasize temporality, succession, and intergenerational dynamics (Fritzsche, 2026). Studies of constrained AI writing systems show that limiting generativity can paradoxically enhance students’ self-regulation, argumentative reasoning, and intellectual autonomy (Konradt et al., 2026). In teacher education, AI-enhanced assessment requires human-in-the-loop practices that preserve interpretive judgment, fairness, and professional responsibility, forms of pedagogical generativity (Fajardo-Ramos et al., 2025). These debates highlight the need to distinguish between algorithmic generativity and human generativity, reinforcing the importance of measuring the latter in educational contexts.

Generativity also appears in participatory educational research, where collaborative inquiry fosters participation, playfulness, passion, and perspicacity, features identified as generative capacities that enable researchers and educators to contribute meaningfully to collective well-being (Van Laren et al., 2013). In multimodal science classrooms, students’ embodied performances generate new forms of knowledge, identity, and solidarity, challenging normative epistemologies and expanding what counts as scientific engagement (Varelas et al., 2024). These examples show that generativity is not only an individual disposition but a collective, relational, and epistemic phenomenon.

Across these diverse literatures, a coherent picture emerges that generativity is a multidimensional construct that integrates motivation, identity, relationality, pedagogy, well-being, and cultural practice. It is central to understanding teachers’ professional lives, yet it remains under-theorized and under-measured in educational research. The Loyola Generativity Scale (LGS) offers a theoretically grounded, widely used instrument for assessing generativity, but its psychometric properties must be validated in specific cultural and professional contexts. Recent evidence suggests that generativity may manifest differently across demographic groups, occupational roles, and cultural settings (Ilea et al., 2025; Hocaoglu and Güvendir, 2025). For teachers, whose work is inherently future-oriented, relational, and identity-forming, validating the LGS is essential for capturing a construct that is both theoretically central and practically consequential.

In the Chilean educational context, where teachers face significant emotional, social, and cognitive demands, generativity may serve as a key psychological resource supporting professional identity, resilience, and well-being. Yet no validated instrument currently exists to measure generativity among Chilean teachers. Establishing the validity, reliability, and factorial structure of the LGS in this population is therefore a critical step toward advancing research on teacher development, mental health, and pedagogical commitment. A strong theoretical grounding in generativity as the central organizing construct of teaching provides the conceptual foundation for this validation study and underscores its relevance to educational psychology and teacher education.

## 2 Methods

### 2.1 Study design

A quantitative, non-experimental, cross-sectional design was used to evaluate the factor structure of the Loyola Generativity Scale (LGS) (McAdams and de St. Aubin, 1992) and to describe the levels of generative engagement among elementary school teachers in Chile. This type of design is consistent with contemporary psychometric research that examines the structural properties of instruments within specific populations without manipulating variables (Ferrando and Lorenzo-Seva, 2017).

### 2.2 Participants

The sample consisted of 430 elementary school teachers from municipal and subsidized private schools in four regions of central and southern Chile: the Santiago Metropolitan Region, Maule, La Araucanía, and Los Ríos. These teachers were drawn from an estimated population of approximately 47.600 elementary school teachers working in these regions during the 2025 academic year, according to administrative records provided by regional education authorities (Ministerio de Educación Centro de Estudios, 2025). Stratified sampling by region and school type was used (Song and Kawai, 2023) to ensure territorial and organizational representation across the main strata of the Chilean school system. Within each stratum, teachers were invited through institutional authorization granted by school principals and local education departments, who distributed the questionnaire internally.

However, the final selection was non-probabilistic and based on voluntary participation, a common procedure in occupational health and school climate studies where access depends on institutional authorization. This approach corresponds to stratified convenience sampling rather than purposive sampling, as no specific teacher characteristics were intentionally targeted beyond the inclusion criteria.

The inclusion criteria were holding a professional degree in elementary education, serving as a teacher in schools (urban or rural), working in municipal or subsidized private schools, and having three or more years of classroom teaching experience.

Following the methodological recommendations of Kanaki and Kalogiannakis (2023), the limitations inherent in non-probability sampling in educational contexts were considered. It was not possible to calculate an accurate response rate because the schools distributed the questionnaire internally; however, the available administrative data did not show systematic differences between participating and non-participating schools.

### 2.3 Procedure

Data collection was conducted using a digital, self-reported, anonymous, and voluntary questionnaire, administered after accepting informed consent. The study complied with the ethical principles of the Declaration of Helsinki and was approved by the Scientific Ethics Committee of the Autonomous University of Chile (Resolution No. 11-25, April 22, 2025).

### 2.4 Instrument

The Loyola Generativity Scale (LGS), developed by McAdams and de St. Aubin (1992), was used to assess generative concern, a core dimension of adult development. The instrument has been extensively validated across diverse contexts and has consistently demonstrated strong internal consistency and robust associations with generative behaviors and autobiographical narratives. As reported by its authors, the LGS “showed strong positive associations with reports of actual generative acts” (McAdams and de St. Aubin, 1992, p. 1003).

The original LGS comprises 20 items organized into two-factor structure consisting of Positive Generativity (17 positively worded items) and Generative Doubts (3 negatively phrased items) (McAdams and de St. Aubin, 1992, p. 1008). These dimensions were theoretically derived from Erikson’s developmental framework and subsequently refined through empirical analyses to maximize internal consistency and convergent validity (McAdams and de St. Aubin, 1992). Responses are recorded on a 4-point Likert scale ranging from “never applies to me” to “very often applies to me”, covering domains such as concern for future generations, productivity, community involvement, and perceived personal impact (McAdams and de St. Aubin, 1992).

For the present study, the LGS was translated and culturally adapted into Spanish following a forward translation process. The translated version was reviewed and validated by a panel of three experts in educational psychology and psychometrics to ensure linguistic clarity, conceptual equivalence, and cultural appropriateness for Chilean teachers. The final adapted version was subsequently administered to the study sample.

### 2.5 Data analysis

The analyses were conducted using FACTOR software, version 12.04.05 (Ferrando and Lorenzo-Seva, 2017), following a robust psychometric procedure for ordinal data.

#### 2.5.1 Sample division using the Solomon method

Since the sample size exceeded 400 cases, the Solomon method (Lorenzo-Seva, 2022) was applied to divide the sample into two equivalent subsamples. This procedure optimizes representativeness and allows evaluation of the stability of the factorial structure across independent partitions of the data. The Solomon split was used exclusively to assess the robustness and replicability of the factor structure, not to estimate the final model. Equivalence between subsamples was verified using the Ratio Communality Index (S), where values close to 1 indicate maximum proportionality.

After confirming the equivalence of the subsamples, the full sample was used for the confirmatory factor analysis (CFA) to maximize statistical power, parameter precision, and model fit accuracy. This sequential approach – stability testing via Solomon followed by CFA on the full dataset – is consistent with recommended psychometric practices for ordinal data and medium-sized samples.

#### 2.5.2 Preliminary item analysis

Univariate descriptive statistics (mean, variance, skewness, and kurtosis) were examined, following criteria for ordinal distributions (Ferrando et al., 2022; Ho and Yu, 2015). Sample adequacy was assessed using the Measure of Sampling Adequacy (MSA) based on the anti-image matrix (Kaiser and Cerny, 1979), the Kaiser-Meyer-Olkin (KMO) index, and Bartlett’s sphericity test. These indicators confirmed the appropriateness of applying factor analysis.

#### 2.5.3 Confirmatory factor analysis (CFA)

The CFA was conducted using polychoric correlations, the Robust Unweighted Least Squares (RULS) method, and factor selection via the Hull method (Lorenzo-Seva et al., 2011). This combination is recommended for ordinal items and prevents the inflation of fit indices commonly observed when maximum likelihood is applied to non-normal data.

To determine the optimal number of factors, we combined multiple criteria:

(a) the Hull method, which balances model fit and parsimony;

(b) the scree plot and Cattell’s (1966) inflection-point criterion;

(c) the Kaiser criterion (eigenvalue > 1); and

(d) theoretical interpretability.

Because the Kaiser criterion tends to overestimate the number of factors (especially when eigenvalues fall marginally above 1) we prioritized the scree plot’s elbow and the Hull method’s optimization curve. All plausible factor structures were then estimated to ensure a comprehensive evaluation.

#### 2.5.4 Model fit assessment

Model fit was assessed using Cumulative Variance Explained (CVE) based on eigenvalues, LOSEFER Chi-square divided by degrees of freedom (χ^2^/df), Root Mean Square Error of Approximation (RMSEA), Adjusted Goodness of Fit Index (AGFI), Goodness of Fit Index (GFI), Comparative Fit Index (CFI), Non-Normed Fit Index (NNFI), and Root Mean Square Residual (RMSR). The interpretation criteria followed the general recommendations of Schermelleh-Engel et al. (2003) and Lorenzo-Seva and Ferrando (2023) for LOSEFER χ^2^/df, and those of Kalkan and Kelecioğlu (2016) for RMSR.

In addition to the global fit indices, a close-fit test and a power analysis were conducted following the LOSEFER correction procedure proposed by Lorenzo-Seva and Ferrando (2023). The close-fit test evaluates whether the Root Mean Square Error of Approximation (RMSEA) is statistically compatible with a close-fitting model (RMSEA < 0.05), while the power analysis quantifies the sensitivity of the test to detect deviations from close fit. Together, these procedures provide complementary and robust evidence for evaluating whether additional factors meaningfully improve the model’s representation of the population structure rather than simply inflating its complexity.

#### 2.5.5 Reliability of factor scores

The following were estimated: Fully-Informative Prior Oblique EAP scores, ORION (Overall Reliability of Fully-Informative Prior Oblique N-EAP scores), and the Factor Determinacy Index (FDI). According to Ferrando and Lorenzo-Seva (2016), ORION assesses the reliability of latent scores (the proportion of true variance captured by the factor score) and is interpreted similarly to omega or composite reliability, with values >0.90 considered excellent. The FDI estimates the correlation between the true (latent) factor and the estimated factor score; a value >0.90 is considered excellent. The approximate relationship ORION ≈ FDI^2^ allowed for the joint interpretation of both indicators (Ferrando and Lorenzo-Seva, 2016).

## 3 Results

The characterization of the teacher sample revealed a broad, heterogeneous distribution across sociodemographic and professional variables. Most participants were women (76%), reflecting the gender composition typical of the Chilean elementary teaching workforce. Age groups were well represented, with the largest segments between 30 and 39 years (35%) and 40–49 years (31%). Teachers worked predominantly in urban schools (76%) and in mixed-funding institutions (69%), although public schools also contributed a substantial proportion of cases (31%). The sample included educators from the Metropolitan (39%), Maule (19%), La Araucanía (26%), and Los Ríos (16%) regions, ensuring territorial diversity across central and southern Chile. Professional experience ranged from early-career teachers to those with more than 20 years in the system, and educational attainment varied from undergraduate degrees (67%) to postgraduate training, including master’s programs (19%). This diversity provided a robust basis for examining generativity patterns across contrasting organizational and territorial contexts (see Table 1). Detailed information is available in the Supplementary material for this article.

**TABLE 1:** Teachers sample characterization.

Sociodemographic variables	Level (number code)	*n*	*n*%
Gender (GND)	Female (1)*	325	76%
	Male (2)	104	24%
	No binary (3)	1	0%
Age level (AGE) (in years)	AGL < 30 (1)	63	15%
	30 ≤ AGL ≤ 39 (2)	151	35%
	40 ≤ AGL ≤ 49 (3)	135	31%
	50 ≤ AGL ≤ 59 (4)	53	12%
	60 ≤ AGL (5)	28	7%
Region (REG)	La Araucanía (LAR)	113	26%
	Los Ríos (LRI)	70	16%
	Maule (MAU)	80	19%
	Metropolitan (MET)	167	39%
Location (LOC)	Rural (1)	104	24%
	Urban (2)	326	76%
Dependence (DEP)	Public (1)	134	31%
	Mixed funding (2)	296	69%
Experience level (EXP) (in years)	EXP < 5 (1)	65	15%
	5 ≤ EXP ≤ 10 (2)	130	30%
	11 ≤ EXP ≤ 15 (3)	110	26%
	16 ≤ EXP ≤ 20 (4)	56	13%
	21 ≤ EXP (5)	69	16%
Education level (EDU)	Ungraduated (1)**	287	67%
	Qualification (2)***	29	7%
	Advanced qualification (3)	6	1%
	Master (4)	82	19%

*The numbers and acronyms in parentheses (°) correspond to the values assigned in the database.

**Ungraduated includes teachers who have not completed postgraduate studies.

***Qualification, including teachers with varying levels of pedagogical specialization at the diploma and postgraduate levels in educational fields.

No missing data were detected in the final dataset, and the factorial analytic approach used in the study reduces the risk of common method bias (CMB) by modeling shared variance at the latent level.

### 3.1 Factorial and reliability findings

The data analysis strategy proceeded sequentially using FACTOR software (version 12.04.05): first, the sample was divided using the Solomon method to assess the stability of the factorial structure across equivalent subsamples; second, preliminary item analyses and matrix adequacy tests were performed to verify compliance with the assumptions required for factor extraction; third, confirmatory factor analyses were conducted using polychoric correlations and robust estimation methods, followed by a comprehensive evaluation of model fit; and finally, the reliability and determinacy of the factor scores were examined to ensure their suitability for subsequent statistical analyses.

#### 3.1.1 Solomon split-sample validation

Given that the total sample consisted of 430 teachers, the Solomon method (Lorenzo-Seva, 2022) was applied to optimally divide the dataset into two equivalent subsamples of 215 cases each. This procedure maximizes representativeness by ensuring that both halves retain the full range of variance present in the original sample. The equivalence between subsamples was evaluated using the Ratio Communality Index (S), which reached 0.9789, an indicator very close to 1 and therefore consistent with a high degree of proportionality between the subsamples. The KMO indices for the first (0.8318) and second (0.8143) subsamples further confirmed their adequacy for subsequent factor analyses (see Table 2).

**TABLE 2:** Solomon method.

Group	*n*	n without missing values	KMO index	S
First Solomon subsample	215	215	0.8318	0.9789
Second Solomon subsample	215	215	0.8143	

The Solomon results were used to verify the stability of the factorial structure across equivalent subsamples. Once structural stability was confirmed, the CFA was conducted on the full sample to obtain more precise parameter estimates and more reliable fit indices. This two-step strategy ensures both robustness (via split-sample validation) and optimal model estimation (via full-sample CFA).

#### 3.1.2 Item-level diagnostics

Univariate descriptive statistics (mean, variance, skewness, and kurtosis) were examined following recommended criteria for ordinal indicators (Ferrando et al., 2022; Ho and Yu, 2015). Item means ranged from 1.644 to 3.456, reflecting adequate variability in generativity levels across the sample. Variances were within acceptable ranges, and most items showed moderate negative skewness and kurtosis values consistent with ordinal response formats. These distributional patterns supported the use of polychoric correlations and robust estimation methods, which reduce bias when items deviate from normality.

Sample adequacy was evaluated using the Measure of Sampling Adequacy (MSA) derived from the anti-image matrix (Kaiser and Cerny, 1979). Consistent with established criteria, MSA values below.50 indicate that an item does not share sufficient common variance with the rest of the item pool and should therefore be removed. Four items of the LGS (GEN5, GEN9, GEN11, and GEN14) fell below this threshold and were excluded from subsequent analyses. The remaining items showed satisfactory MSA values, confirming their suitability for factor extraction (see Table 3).

**TABLE 3:** Univariate descriptive statistical analysis.

Variables	Mean		Variance	Skewness	Kurtosis	MSA for 20 items		MSA for 16 items	
	Statistic	CI 95%	Statistic	Statistic	Statistic	Normed	CI 95% (Bootstrap)	Normed	CI 95% (Bootstrap)
GEN1	3.360	(3.26; 3.46)	0.621	−1.105	0.612	0.83866	(0.572; 0.870)	0.85801	(0.622; 0.885)
GEN2	1.756	(1.65; 1.86)	0.701	0.962	0.294	0.78931	(0.599; 0.832)	0.76354	(0.594; 0.813)
GEN3	3.142	(3.03; 3.25)	0.805	−0.747	−0.368	0.90153	(0.716; 0.916)	0.92635	(0.760; 0.938)
GEN4	3.028	(2.92; 3.13)	0.725	−0.416	−0.716	0.88471	(0.758; 0.906)	0.88392	(0.772; 0.912)
GEN5	2.209	(2.09; 2.33)	0.882	0.400	−0.702	0.71468*	(0.430; 0.804)	–	–
GEN6	3.144	(3.05; 3.24)	0.630	−0.599	−0.277	0.92268	(0.800; 0.931)	0.92744	(0.827; 0.939)
GEN7	3.398	(3.30; 3.49)	0.612	−1.091	0.332	0.8717	(0.735; 0.898)	0.87158	(0.750; 0.905)
GEN8	2.500	(2.37; 2.63)	1.041	0.026	−1.113	0.82692	(0.673; 0.861)	0.83386	(0.705; 0.871)
GEN9	2.093	(1.97; 2.22)	0.991	0.479	−0.874	0.58933*	(0.341; 0.669)	–	–
GEN10	2.602	(2.49; 2.72)	0.853	−0.107	−0.829	0.86568	(0.756; 0.889)	0.87627	(0.767; 0.900)
GEN11	2.695	(2.55; 2.84)	1.407	−0.215	−1.478	0.76939*	(0.475; 0.819)	–	–
GEN12	3.456	(3.36; 3.55)	0.564	−1.202	0.611	0.88020	(0.747; 0.896)	0.88054	(0.765; 0.905)
GEN13	1.891	(1.78; 2.00)	0.832	0.770	−0.284	0.81238	(0.653; 0.847)	0.76821	(0.609; 0.823)
GEN14	2.067	(1.93; 2.20)	1.179	0.521	−1.092	0.70441*	(0.467; 0.756)	–	–
GEN15	1.644	(1.54; 1.75)	0.713	1.191	0.609	0.80977	(0.667; 0.844)	0.79344	(0.651; 0.841)
GEN16	3.267	(3.16; 3.38)	0.777	−0.916	−0.209	0.91144	(0.771; 0.928)	0.94377	(0.842; 0.948)
GEN17	3.235	(3.13; 3.34)	0.687	−0.879	0.093	0.91432	(0.816; 0.926)	0.91466	(0.828; 0.930)
GEN18	3.037	(2.92; 3.16)	0.910	−0.541	−0.843	0.84390	(0.644; 0.878)	0.85397	(0.678; 0.893)
GEN19	2.995	(2.89; 3.10)	0.670	−0.476	−0.330	0.89042	(0.770; 0.909)	0.89684	(0.801; 0.919)
GEN20	2.758	(2.64; 2.87)	0.881	−0.178	−0.932	0.84804	(0.725; 0.870)	0.85006	(0.751; 0.876)

*Items proposed to be removed based on MSA: GEN5, GEN9, GEN 11, and GEN14.

The adequacy of the polychoric correlation matrix was confirmed through multiple indicators. The determinant of the matrix was 0.0019, a value sufficiently distant from zero to indicate the absence of multicollinearity problems and the presence of meaningful shared variance among items. Bartlett’s test of sphericity was highly significant, χ^2^(120) = 2645.4, *p* < 0.001, demonstrating that the correlation matrix departs from the identity matrix and is therefore suitable for factor extraction.

The Kaiser–Meyer-Olkin (KMO) index was 0.872, indicating good sampling adequacy. The bootstrap 95% confidence interval for the KMO (0.831–0.905) further confirmed the stability and robustness of this estimate. Together, these indicators show that the data structure is appropriate for applying factor analysis using polychoric correlations and robust estimation methods.

#### 3.1.3 Confirmatory factor structure

The confirmatory factor analysis (CFA) was conducted using polychoric correlations, the Robust Unweighted Least Squares (RULS) estimator, and factor retention guided by the Hull method (Lorenzo-Seva et al., 2011). To explore all plausible factorial solutions, we examined the scree plot and applied Cattell’s (1966) inflection-point criterion.

**FIGURE 1:**
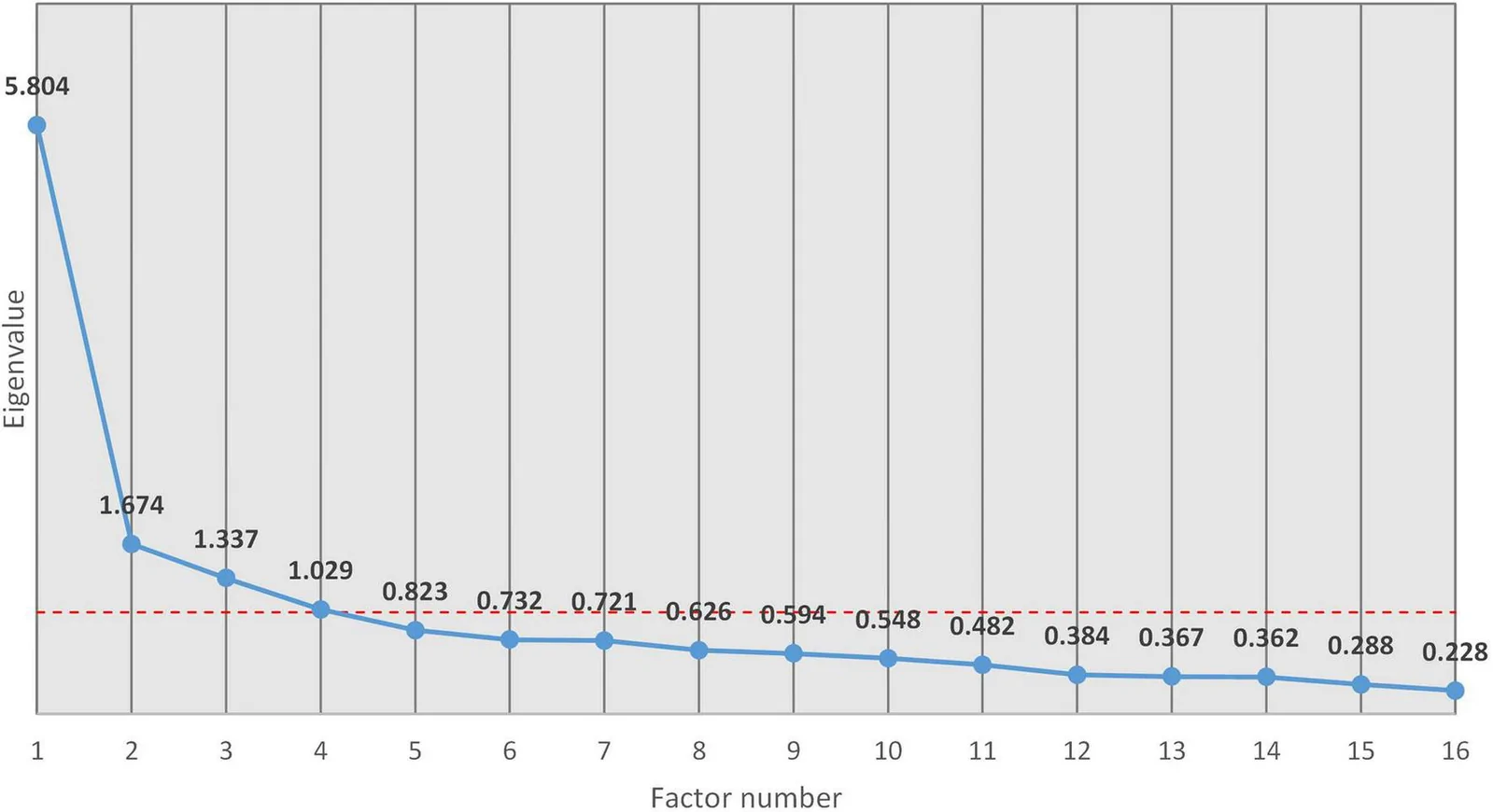
Scree plot. Elbow point indicated in red and clipping line (eigenvalue = 1) in red dashed line.

As shown in Figure 1, the scree plot displays a clear elbow after the third factor, with the first three eigenvalues (5.804, 1.674, and 1.337) exceeding the Kaiser threshold (eigenvalue = 1). Although the fourth eigenvalue (1.029) lies marginally above the clipping line, its position does not alter the inflection pattern and is characteristic of the Kaiser criterion’s tendency to over-extract factors. The slope between factors 3 and 4 shows no meaningful discontinuity, and the fourth factor contributes minimal unique variance.

The Hull method also selected the three-factor solution as the optimal balance between fit and parsimony, whereas the four-factor model showed only trivial improvements in fit at the cost of reduced theoretical coherence and factor interpretability.

This analytic strategy is recommended for ordinal indicators and prevents the inflation of fit indices commonly observed when maximum likelihood is applied to non-normal data.

Taken together, the scree plot, eigenvalue distribution, Hull optimization, and theoretical considerations converge to support retaining a three-factor solution.

#### 3.1.4 Model fit performance

Comparisons of alternative factorial solutions revealed clear differences in model performance. As summarized in Table 4, the one-factor model exhibited the weakest fit, with CVE = 36.3% and χ^2^/df = 4.431, alongside RMSEA and RMSR values above the recommended thresholds. The two-factor model improved substantially (CVE = 46.7%; χ^2^/df = 3.137), reaching acceptable levels across most indices. However, the three-factor solution demonstrated the best overall fit, with CVE = 55.1%, χ^2^/df = 1.792, RMSEA = 0.043, AGFI = 0.985, GFI = 0.991, CFI = 0.989, NNFI = 0.983, and RMSR = 0.041, all within the “good fit” range.

**TABLE 4:** Validation and reliability versus parameters (Schermelleh-Engel et al., 2003).

Model	Sample	CVE	MIF	χ^2^/df^+^	RMSEA	AGFI	GFI	CFI	NNFI	RMSR^++^
Proposed model (One-factor)	430	36.3%	16*	4.431	0.089 (0.087; 0.092)	0.947** (0.917; 0.953)	0.954** (0.928; 0.959)	0.937 (0.929; 0.943)	0.927 (0.918; 0.934)	0.0909 (0.083; 0.093)
Proposed model (Two-factor)	430	46.7%	3*	3.137	0.071* (0.068; 0.074)	0.968** (0.945; 0.971)	0.976** (0.959; 0.978)	0.966* (0.961; 0.971)	0.955* (0.947; 0.960)	0.0655* (0.061; 0.066)
Proposed model (Three-factor)	430	55.1%	3*	1.792	0.043** (0.041; 0.046)	0.985** (0.976; 0.992)	0.991** (0.985; 0.995)	0.989** (0.984; 0.990)	0.983** (0.974; 0.985)	0.0410** (0.030; 0.030)
Proposed model (Four-factor)	430	61.5%	2	1.446	0.032** (0.029; 0.035)	0.990** (0.978; 0.990)	0.995** (0.989; 0.995)	0.995** (0.992; 0.997)	0.991** (0.985; 0.995)	0.0306* (0.021; 0.021)
Adopted thresholds	≥200	**	NR	[0, 2]	[0.00, 0.05]	[0.90, 1.00]	[0.95, 1.00]	[0.97, 1.00]	[0.97, 1.00]	[0.00, 0.05)^++^
		*	≥ 3	(2, 3]	(0.05, 0.08]	[0.85, 0.90)	[0.90, 0.95)	[0.95, 0.97)	[0.95, 0.97)	[0.05, 0.08]^++^

NR: not reported.

**Good fit;

*Acceptable fit.

^+^LOSEFER empirically corrected Chi-square.

^++^indicated in Kalkan and Kelecioğlu (2016).

A four-factor model was also computed and reported for transparency. Although its fit indices were slightly higher, this configuration lacked theoretical coherence, produced less stable factor loadings, and did not align with the scree plot or the Hull method. The fourth factor was weak, poorly defined, and contributed little unique variance, consistent with an over-extraction artifact.

Therefore, the three-factor solution was retained as the most parsimonious, theoretically meaningful, and empirically robust representation of generativity in Chilean teachers. Table 4 presents a concise comparison of the four tested models alongside the adopted thresholds.

The three-factor solution displayed a clear and theoretically coherent structure. As shown in Table 5, Factor 1 (Generative Inefficacy) grouped the reverse-coded items reflecting feelings of low impact, lack of usefulness, and diminished generative self-efficacy (GEN2, GEN13, GEN15), with standardized loadings ranging from 0.617 to 0.801. Factor 2 (Generative Action) captured concrete prosocial behaviors and commitments – such as creativity, productivity, teaching, and community engagement – with loadings between 0.426 and 0.745 across items GEN1, GEN6, GEN7, GEN12, GEN16, GEN17, GEN18, and GEN19. Factor 3 (Generative Legacy) represented motivations related to symbolic permanence and societal contribution, with strong loadings for GEN8, GEN10, and GEN20 (0.605–0.735).

**TABLE 5:** Confirmatory factor analysis for three factors.

Variable (Item)	Factor 1 (F1)	Factor 2 (F2)	Factor 3 (F3)
GEN1		0.502 (0.332; 0.688)
GEN2	0.617 (0.475; 0.738)	
GEN6		0.530 (0.386; 0.654)
GEN7		0.639 (0.491; 0.781)
GEN8			0.735 (0.553; 0.946)
GEN10			0.605 (0.482; 0.723)
GEN12		0.745 (0.605; 0.880)	
GEN13	0.664 (0.524; 0.787)		
GEN15	0.801 (0.685; 0.955)		
GEN16		0.580 (0.467; 0.714)	
GEN17		0.706 (0.593; 0.825)	
GEN18		0.426 (0.273; 0.582)	
GEN19		0.596 (0.461; 0.717)	
GEN20			0.693 (0.540; 0.846)
**Factor**	**F1**	**F2**	**F3**
F1	1.000	1.000	1.000
F2	−0.340 (−0.460; −0.228)		
F3	−0.295 (−0.478; −0.194)	0.448 (0.370; 0.538)	

The interfactor correlations indicated that the three dimensions were related but non-redundant: Generative Inefficacy correlated negatively with both Generative Action (*r* = –0.340) and Generative Legacy (*r* = –0.295), whereas Generative Action and Generative Legacy showed a moderate positive association (*r* = 0.448). This pattern supports the interpretation of three distinct yet interconnected facets of generativity within the teaching profession.

Overall, the magnitude and consistency of the loadings, together with the interfactor structure, provide strong evidence for a differentiated three-factor configuration of generativity among Chilean elementary school teachers (see Table 5).

Additionally, to further evaluate the quality and stability of the three-factor solution, a close-fit test and a power analysis were conducted after applying the LOSEFER correction (Lorenzo-Seva and Ferrando, 2023). The Root Mean Square Error of Approximation (RMSEA) was 0.043, and the close-fit test yielded a high probability (*p* = 0.83) for RMSEA < 0.05, indicating that the model achieves a close fit in the population. The power analysis showed β > 0.99 for distinguishing between RMSEA = 0.05 (H0) and RMSEA = 0.08 (H1), demonstrating that the sample size (*N* = 430) provides more than sufficient sensitivity to detect deviations from close fit. Together, these results reinforce the robustness of the three-factor model and confirm that its excellent fit indices are not sample-specific artifacts but reflect a stable underlying structure.

#### 3.1.5 Reliability metrics

The reliability of the estimated factor scores was satisfactory across the three latent dimensions. As shown in Table 6, ORION (Overall Reliability of Fully-Informative Prior Oblique N-EAP scores) values ranged from 0.795 to 0.863, indicating adequate to very good reliability of the factor score estimates. The Factor Determinacy Index (FDI), which quantifies the correlation between the true latent factor and its estimated score, ranged from 0.892 to 0.929, with values above 0.90 considered excellent. The squared determinacy values (FDI^2^) were consistent with the corresponding ORION coefficients, as expected from their theoretical relationship.

**TABLE 6:** Relaibility analysis for three factors.

Factor	Variance	ORION	Interpretation ORION	FDI	Interpretation FDI	FDI^2^
F1	1.704	0.795	0.70–0.80, adequate–high	0.892	0.80–0.90, very good	0.796
F2	3.582	0.863	0.80–0.90, very good	0.929	>0.90, excellent	0.863
F3	2.059	0.824	0.80–0.90, very good	0.908	>0.90, excellent	0.825

Regarding the variance explained, the sum of the rotated factor variances was 7.345. When divided by the 14 retained items, the three factors jointly accounted for approximately 52.5% of the total variance, indicating that a substantial proportion of the common variance in generativity is captured by this trifactorial solution. Taken together, these results show that the factor scores are both reliable and well-determined, supporting their use in subsequent analyses (see Table 6).

## 4 Discussion

The findings of this study provide robust evidence for the factorial structure and psychometric properties of the Loyola Generativity Scale (LGS) among Chilean elementary school teachers. Item refinement based on MSA criteria led to a more parsimonious and conceptually coherent solution. In contrast to the original two-factor structure reported by McAdams and de St. Aubin (1992) – Positive Generativity and Generative Doubts – the present study identified a differentiated three-factor configuration. This expansion is theoretically meaningful: while the original LGS captures broad motivational tendencies toward generativity, the teaching profession appears to activate additional, context-specific facets linked to educators’ daily responsibilities, relational demands, and symbolic commitments

The three-factor solution exhibited a clear and theoretically coherent structure. Generative Inefficacy grouped reverse-coded items reflecting feelings of low impact and diminished generative self-efficacy. This dimension does not appear explicitly in previous LGS validations, but it resonates with research showing that teachers perceived effectiveness strongly shapes their generative motivations (Faßbender et al., 2019). Generative Action captured concrete prosocial behaviors (creativity, productivity, teaching, and community engagement), aligning with the original Positive Generativity factor. Generative Legacy represented motivations oriented toward symbolic permanence and societal contribution, partially overlapping with the original conceptualization of generative concern but emerging here as a distinct motivational domain.

This structure both overlaps with and extends previous findings. Studies conducted in collectivist or community-oriented contexts (Hofer et al., 2008; Marushima and Arimitsu, 2007) have shown that generativity can adopt multidimensional expressions, but none identified a factor analogous to Generative Inefficacy. The emergence of this factor in Chile may reflect contextual pressures such as high emotional demands, accountability policies, and persistent inequalities in the school system, which can heighten teachers’ doubts about their long-term impact. In contrast, the strong and coherent loadings of Generative Action and Generative Legacy align with international evidence showing that generativity is closely tied to prosocial commitment and symbolic continuity across cultures.

The interfactor correlations further illuminate the dynamics of generativity in teaching. Generative Inefficacy correlated negatively with both Generative Action and Generative Legacy, suggesting that doubts about one’s impact may inhibit both concrete prosocial behaviors and symbolic motivations. Meanwhile, the moderate positive correlation between Generative Action and Generative Legacy indicates that teachers who engage actively in prosocial practices also tend to articulate broader societal purposes. This pattern suggests that generativity in teaching is not merely a personal disposition, but a relational and contextual process shaped by school culture, community expectations, and professional identity.

High ORION and FDI values, along with the model’s stability across subsamples, reinforce the scale’s internal validity and relevance for educational contexts. The elevated levels of generativity observed in the sample align with research showing that caring, teaching, and community-oriented professions tend to exhibit high generative concern (Ilea et al., 2025; Hofer et al., 2008). However, the differentiated structure found here suggests that generativity in teachers is more complex than previously assumed, integrating both motivational strengths and perceived inefficacies. This nuance represents an improvement over earlier validations, which tended to treat generativity as a homogeneous construct.

### 4.1 Theoretical implications

The findings strengthen the understanding of generativity as a central construct in teachers’ professional identity. The psychometric evidence supports the LGS as a valid measure of generative concern, enabling theoretical models that integrate generativity with key educational variables such as pedagogical commitment, prosocial motivation, and well-being.

The factorial stability found here is consistent with longitudinal studies showing high rank-order stability of generativity in adulthood (Einolf, 2014). Cross-cultural research (Hofer et al., 2008; Marushima and Arimitsu, 2007) also indicates that although generativity may vary in expression across sociocultural contexts, its conceptual structure remains stable. The Chilean context adds a unique layer: teachers operate in environments marked by social inequality, strong community ties, and high expectations for socioemotional support, which may amplify both generative action and generative doubts.

The study also aligns with literature linking generativity to agency and communion motivations (Faßbender et al., 2019; Ilea et al., 2025). For teachers, agency manifests in the creation of pedagogical practices, while communion emerges in student care and support, consistent with the view of generativity as a mature form of psychosocial functioning (Kotre, 1984; McAdams and de St. Aubin, 1992). The three-factor structure identified here provides a more granular lens to examine how agency and communion interact within teaching roles.

### 4.2 Practical implications

A validated version of the LGS for Chilean teachers offers concrete opportunities for educational practice. The scale can be used to assess generative concern within teaching teams, informing interventions to strengthen purpose, prosocial motivation, and alignment with the educational mission.

Generativity may also serve as an indicator in teacher training programs, particularly in socioemotional competencies, pedagogical leadership, and mentoring – dimensions associated with well-being, resilience, and professional commitment (Aparisi et al., 2020; Kim and Youn, 2002; Sandoval-Obando et al., 2026b,2026a). The identification of Generative Inefficacy is especially relevant for designing interventions aimed at reducing burnout and strengthening teachers’ perceived impact.

Finally, the validated scale provides a useful tool for research exploring how generativity relates to pedagogical practices, school climate, intergenerational relationships, and mentoring processes (Ilea et al., 2025; Hofer et al., 2008). The three-factor structure enables more targeted analyses of how different facets of generativity influence classroom dynamics and professional development.

### 4.3 Limitations

Non-probabilistic sampling limits generalizability, and the cross-sectional design prevents causal inference. The removal of four items reduces comparability with international studies using the full LGS (Marushima and Arimitsu, 2007; Einolf, 2014). Self-report measures may also introduce social desirability bias.

### 4.4 Future research

Future studies should examine factorial invariance across teacher subgroups, investigate longitudinal trajectories of generativity (Einolf, 2014), and integrate generativity into broader explanatory models including self-efficacy, pedagogical leadership, mentoring, and socioemotional competencies (Aparisi et al., 2020; Hofer et al., 2008; Ilea et al., 2025). Further research should also explore how generativity manifests in concrete pedagogical practices and teacher interactions. The three-factor structure identified here opens new avenues for studying how generative doubts, actions, and legacy motivations evolve across teachers’ careers and respond to institutional changes.

## 5 Conclusion

This study provides robust psychometric evidence supporting the validity and structural coherence of the Loyola Generativity Scale among Chilean primary-school teachers. While the original LGS was empirically structured around two dimensions; Positive Generativity and Generative Doubts (McAdams and de St. Aubin, 1992), the present findings reveal a differentiated three-factor configuration comprising Generative Inefficacy, Generative Action, and Generative Legacy. This shift from a bifactorial to a three-dimensional structure represents a substantive contribution: it indicates that generativity, when expressed within the teaching profession, adopts additional facets not captured in the original instrument.

The emergence of a third factor, Generative Inefficacy, suggests that teachers’ generative functioning is shaped not only by prosocial behaviors and symbolic motivations but also by perceived limitations in their impact. This dimension appears to be context-dependent and may reflect structural and emotional pressures characteristic of the Chilean educational system, such as high socioemotional demands, accountability expectations, and persistent inequalities. In contrast, Generative Action and Generative Legacy align with the original LGS dimensions, but their separation into distinct factors provides a more nuanced understanding of how teachers enact and conceptualize their contributions to future generations.

Overall, the refined instrument demonstrated strong factorial stability, high reliability, and theoretical coherence, confirming that generativity is a measurable, multidimensional psychological resource within the teaching profession. By expanding the LGS from two to three empirically grounded dimensions, this study improves upon previous validations and offers a more context-sensitive model of generativity in educators.

The results advance the assessment of generativity in Latin American contexts and provide a methodologically sound foundation for future research. The validated scale can support studies examining the role of generativity in teacher well-being, professional development, intergenerational contributions, and educational outcomes. Future research should evaluate measurement invariance across relevant subgroups, explore longitudinal trajectories of generativity throughout the teaching career, and integrate this construct into broader explanatory models of teacher development and educational effectiveness.

## Data Availability

The original contributions presented in the study are included in the article/Supplementary material, further inquiries can be directed to the corresponding authors.
